# Sugarcane fibre may prevents hairball formation in cats*

**DOI:** 10.1017/jns.2014.27

**Published:** 2014-09-25

**Authors:** Bruna A. Loureiro, Guilherme Sembenelli, Ana P. J. Maria, Ricardo S. Vasconcellos, Fabiano C. Sá, Nilva K. Sakomura, Aulus C. Carciofi

**Affiliations:** 1 College of Agrarian and Veterinarian Sciences (FCAV), São Paulo State University (UNESP), Via de Acesso Professor Paulo Donato Castellane, Jaboticabal 14.884-900, SP, Brazil; 2 State University of Maringá – UEM, Av. Colombo, 5.790, Maringá 87020-900, PR, Brazil

**Keywords:** Cellulose, Feline nutrition, Insoluble fibre, Trichobezoars, CO, control diet

## Abstract

Hair ingested by licking during cat grooming can eventually coalesce into solid masses in cat gastrointestinal tract. It is believed that dietary fibre might reduce formation of these trichobezoars (hairballs). The effects of two insoluble fibre sources added to kibble diets were evaluated with respect to trichobezoar faecal excretion. Thirty-two cats and four diets were used in a randomised block design: a control diet without additional fibre, 10 % added sugarcane fibre, 20 % added sugarcane fibre or 10 % added cellulose. Animals were fed for 42 d and during three separate periods (days 15–17, 25–27 and 40–42), the cats were housed individually in metabolic cages and their faeces were totally collected. The faeces were evaluated and the trichobezoars were isolated and classified into small (<1 cm), medium (1·1–2 cm) or large (>2·1 cm). Means were evaluated by repeated measures ANOVA and contrasts (*P* < 0·05). Cats fed sugarcane fibre shown a linear reduction of small and medium trichobezoar excretion (number per cat per day; *P* = 0·004) as well as a reduction in trichobezoar mass excretion (mg per cat per day; *P* < 0·01). The control group showed increased faecal excretion of large trichobezoars (*P* = 0·003), which were not present in the high sugarcane fibre group (*P* < 0·006). No effect of cellulose was observed for any evaluated trait. Therefore, long fibres (sugarcane fibre) may cause greater peristaltic stimulation, increasing the propulsion of hair through the gut, but further research is needed to validate this mechanism. In conclusion, sugarcane fibre reduced faecal hairball elimination in cats, which may have clinical applications for the prevention of health problems related to trichobezoars.

The formation of hairballs, or trichobezoars, in cats is a result of the daily hygiene routine of grooming during which the animals lick and ingest their own hair. Some cats have been observed to spend up to one-third of their awake time grooming. The ingested hair is moved through stomach and intestines by peristalsis and when hair becomes entangled it can coalesce into a solid mass^(^^1^^)^. Some cats can regurgitate the hairball to eliminate it; however, some trichobezoars can accumulate into large masses that potentially may cause dangerous gastrointestinal obstructions^(^^2^^,^^3^^)^.

At present, the literature about the prevalence and predisposing factors for hairball formation is limited. Some studies have suggested hairball formation and health risks are associated with some breeds. Specifically, it has been reported that feline hairball formation may be associated with reduced intestinal motility occurring during prolonged fasting^(^^4^^)^ and with long-haired cats or cats that display frequent grooming and swallowing of hair^(^^5^^)^.

It has been suggested that both protein and fibre intake may be involved in hairball formation^(^^2^^)^. Hair and trichobezoars in faeces were associated with low-fibre intake in ruminants^(^^6^^)^. In rabbits, the main risk factors for trichobezoar formation are low-fibre intake, animals with long hair, nutritional deficiencies, stress and boredom^(^^7^^)^. The importance of fibre type (soluble or insoluble) and size (length and width) on the prevention of hairball formation has also been studied in rats^(^^2^^)^. Few published studies directly demonstrate the correlation of these factors with hairball formation in cats. In a 2-week study on the use of a palatable chew containing psyllium (a source of soluble fibre) and slippery elm (used in herbal medicine as an emollient, expectorant and diuretic) there was a 29 % reduction in clinical signs associated with hairballs (coughing, retching and vomiting) although there was no significant reduction in actual vomiting^(^^8^^)^. In another study, cats fed a diet with 4 % added cellulose showed a reduction in the severity of clinical symptoms of hairballs. The total incidence of vomiting, retching and coughing was reduced by 79, 91 and 70 %, respectively, in comparison with the placebo-controlled food, a regular diet without added fibre^(^^9^^)^.

The purpose of the present study was to evaluate the effect of two insoluble, non-fermentable fibres (sugarcane fibre and purified cellulose) on trichobezoar formation and faecal elimination in cats.

## Material and methods

The experiment was conducted at the Laboratory of Research on Nutrition and Nutritional Diseases of Dogs and Cats, UNESP, Jaboticabal, Brazil. All procedures were approved by the Ethics and Animal Welfare Committee (Protocol 20.481/10).

### Animals and study design

Thirty-two mixed breed cats were used in the study. The average age was 6·0 (sem 0·21) years old with an average weight of 4·2 (sem 0·02) kg and body composition score of 5, in a nine-point scale. In total fifteen males and seventeen females, all of which were neutered or spayed, were used. Good health status was confirmed prior to beginning the study.

The study followed a randomised block design with four diets and two blocks of sixteen animals each. There were four animals per diet in each block, therefore there was a total of eight animals per diet. The cats were divided into the block and diet groups according to body weight, sex and hair coat type (short hair or long hair). Each block lasted 42 d. Collection of faeces was performed during three periods that each lasted 3 d: days 15–17, days 25–27 and days 40–42. During the faecal collection periods the cats were housed individually in metabolic cages measuring 0·80 × 0·80 × 1·0 m, and all faecal matter was collected at least two times a day, weighed, pooled by cat and stored frozen (−20°C) for future analysis. Except during the periods of faecal collection, when the cats were restricted to their metabolic cages, the animals were housed from 16·00 to 08·00 h in individual metabolic cages where they were presented with the experimental foods, and from 08·00 to 16·00 h the cats were allowed to roam free in a collective cattery of 50 m^2^ to exercise and socialise. Throughout the study, a 12 h dark:12 h light cycle was maintained.

The cats were fed individually calculated amounts of food. Initially, the amount was defined according to the energy requirements for cat maintenance, estimated as 418 kJ metabolisable energy per kg^0·67^, where food metabolisable energy was estimated from the diets' chemical compositions^(^^10^^)^. Food consumption was recorded daily and the cats were weighed weekly after which the amount of food provided was adjusted to maintain constant body weight during the study. Water was available *ad libitum*.

### Diets

Four diets with similar protein and fat contents were formulated according to the nutritional recommendations for feline maintenance by the European Pet Food Industry Federation^(^^11^^)^. The diets were based on corn, poultry by-product meal, broken rice, corn gluten meal and poultry fat. The only variation in the diets was the level of insoluble fibre. The control diet (CO) contained no added fibre and contained 32 % protein, 12 % fat and 13 % dietary fibre. The other three diets were based on the CO diet with added fibre that replaced the corn. The 10 % sugarcane fibre diet contained 32·7 % protein, 12·2 % fat and 19·9 % dietary fibre. The 20 % sugarcane fibre diet contained 31 % protein, 12 % fat and 27 % dietary fibre. The 10 % cellulose diet contained 32 % protein, 11·8 % fat and 20·1 % dietary fibre. The DM, crude protein and acid-hydrolysed fat of the diets were analysed using the standard methods^(^^12^^)^. Total dietary fibre was measured according to the method of Prosky *et al.*^(^^13^^)^. The sugarcane fibre (Vit2be Fiber, Dilumix) is a source of insoluble, non-fermentable fibre with an average length of 188 (sem 0·31) µm. The purified cellulose (Minérios Ouro Branco Ltda) has a mean length of 112 (sem 0·3) µm.

The diets were processed in the extruder facility of the College of Agrarian and Veterinarian Sciences, São Paulo State University. The ingredients were mixed and ground in a hammer mill (Model 4, D'Andrea) fitted with a 0·8 mm screen sieve. The diets were extruded under identical processing conditions in a single-screw extruder (Mab 400S, Extrucenter), with an average extrusion capacity of 150 kg/h. A laboratory-scaled complete extrusion system was used which has the same components and standards of operation as extruders for commercial production. The manufacturing process was controlled by adjusting the kibble density between 350 and 370 g/l (as-is basis) every 15 min to ensure consistent cooking and kibble quality (size and expansion). The extruder pre-conditioning temperature was kept above 90°C. Water, steam, screw speed and food flux were adjusted according to diet formulation, and the extrusion temperature varied between 125 and 135°C. Starch gelatinisation degree was adequate in all diets, with values >80 %.

### Trichobezoar quantification in cat faeces

**Fig. 1.**
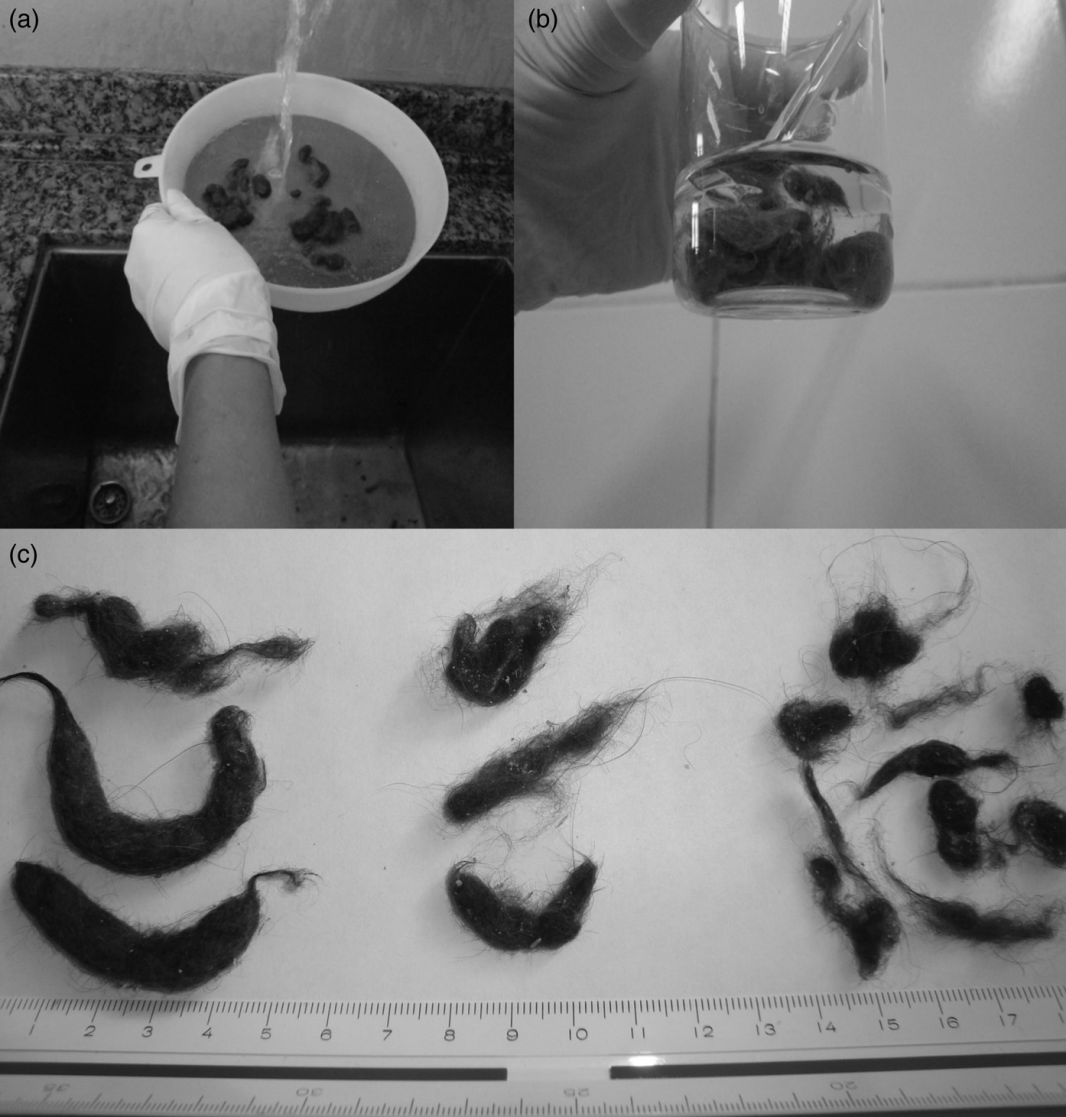
(a) Washing the faeces in tap water to remove faecal material from trichobezoars. (b) Cleaning dry trichobezoars in diethyl ether, to remove residual faecal material. (c) Picture of large, medium and small cat trichobezoars, obtained from faeces.

We were unable to find published studies that measured trichobezoars in cat faeces. Thus we developed the following protocol based on our observations of cat faecal traits. Stored, frozen faeces were thawed and a 2 g sample was sectioned off for the DM measurement. The remaining faecal material was washed with tap water over a sieve with 0·8 mm screen size and gently washed until all faecal material was removed and only hair remained. The remaining trichobezoars were collected and dried in a forced air oven at 55°C for 24 h (320-SE, FANEM), and washed in a 1:1 (v/v) solution of ethylic diethyl ether and petroleum diethyl ether until all foreign materials were removed from the sample. The isolated trichobezoars were then classified according to size in small (>1 cm), medium (1·1–2 cm) or large (<2·1 cm), counted and weighed (Fig. 1).

### Statistical analysis

The experiment was analysed as a randomised block design, with two blocks (periods), four diets and eight cats per diet. The data were analysed using the General Linear Model procedures of the Statistical Analysis Systems statistical software package version 9·0 (SAS Institute). The experimental unit was one cat. The model sums of squares were separated into block, diet and animal effects, and diet × animal interactions. Repeated measures ANOVA with two inter-animal factors (diet and period) and one intra-animal factor (day of sampling) was the statistical method chosen to evaluate the effects of diet and time on trichobezoars. Pairwise means comparisons were also made using Tukey's test when the ANOVA *F* test results were statistically significant. If the diet × time interaction was not significant, the mean of the three times of evaluation was used to compare the diet effects, and the results were submitted to polynomial contrasts (evaluation of the inclusion levels of sugarcane fibre) and orthogonal contrasts (comparison of sugarcane fibre (CO + 10 % sugarcane fibre +20 % sugarcane fibre) *v.* 10 % cellulose). Values of *P* < 0·05 were considered significant. All data were found to comply with the assumptions of ANOVA models.

## Results

All cats adequately consumed their diets and maintained near constant body weight during the experiment (data not shown). All cats produced adequately formed faeces and there were no episodes of soft faeces or diarrhoea. No interaction for time and diet was observed for small or medium trichobezoars in the faeces, so the mean values of all three periods were evaluated (Table 1). Sugarcane fibre inclusion in the diets induced a linear reduction of hairballs present in the faeces, both by mg per cat per day and number per cat per day (*P* < 0·01). Faecal trichobezoars presence in mg per g of faecal DM was numerically reduced with sugarcane fibre inclusion, although it was not significantly different from CO. The number of small- and medium-sized trichobezoars was reduced linearly with sugarcane fibre addition (*P* < 0·01). Cellulose addition, on the other hand, did not affect faecal trichobezoar presence. In the orthogonal contrast, the cats fed cellulose expelled more medium trichobezoars in their faeces than cats fed sugarcane fibre (*P* = 0·047).

**Table 1.** Trichobezoars isolated from the faeces of cats fed kibble diets with different amounts and sources of fibre

	Diets					Contrasts^†^ (*P* value)		
Item	CO	SF10	SF20	CEL10	sem*	Linear	Quadratic	Orthogonal
Trichobezoars								
Time × diet (*P* > 0·05)^‡^								
mg per cat per day	111·0	101·0	87·0	143·0	20·0	0·008	NS	NS
Number per cat per day	6·6	4·4	3·8	6·8	0·6	0·004	NS	0·04
mg per g of faeces DM	11·0	8·5	4·7	8·4	1·6	NS	NS	NS
Number of small per cat per day^§^	5·0	3·0	3·0	4·9	0·5	0·007	NS	NS
Number of medium per cat per day^§^	1·2	1·1	0·6	1·5	0·2	0·048	NS	0·047
Time × diet (*P* = 0·027)^‖^								
Number of large per cat per day^§^						*P* value		
Days 15–17	0·37^b^	0·47	0·35	0·57	0·12	NS		
Days 25–27	0·40^ab^	0·03	0·2	0·48	0·06	NS		
Days 40–42	0·80^Aa^	0·33^AB^	0·00^B^	0·19^AB^	0·08	0·006		
*P* value	0·003	NS	NS	ns				

CO, control diet, without supplemental fibre; SF10, supplemented with 10 % sugarcane fibre; SF20, supplemented with 20 % sugarcane fibre; CEL10, supplemented with 10 % cellulose.

^a,b^Means in the column not sharing a common lower case differ (*P* < 0·05).

^A,B^Means in the row not sharing a common upper case differ (*P* < 0·05).

*Standard error of the mean, *n* 8 cats per diet.

^†^Linear or quadratic effect of sugarcane fibre inclusion. Orthogonal contrast = CEL10 *v.* CO + SF10 + SF20.

^‡^No time × diet interaction (*P* > 0·05), results are the mean of the times of evaluation.

^§^Tricobezoar sizes: small, <1 cm; medium, from 1·1 to 2 cm; large, >2·1 cm.

^‖^Time × diet interaction (*P* < 0·05), results compared considering the time and diet effects.

Regarding large faecal trichobezoars, there was a significant time *v.* diet interaction (*P* = 0·027). Cats fed CO diet exhibited an increased elimination of large trichobezoars during the experimental period (*P* = 0·003). From days 40 to 42 of the evaluation period, no large trichobezoars were detected in the faeces of cats fed the diet contained 20 % sugarcane fibre, meanwhile cats fed the CO diet eliminated 0·8 trichobezoars per cat per day (*P* = 0·006).

## Discussion

As previously reported in other animal species, cats also respond to dietary supplementation of long fibres to reduce hairballs. Although not directly measured during the study, no evidence of reduced hair grooming or licking was observed for any of the diets. During the experiment, all cats exhibited normal hair coat characteristics and behaviour without any evidence of reduced hygiene activity. So, it is probable that the same amount of hair were swallowed and excreted by the cats fed all treatments.

The most probable reason for the reduced faecal trichobezoars load after the consumption of kibble diets supplemented with sugarcane fibre is that the fibre altered the kinetics of peristalsis. Altered peristalsis may reduce hair entanglement and hairball formation, resulting in faecal elimination of untangled loose hair. A previously proposed mechanism^(^^9^^)^ for the reduction of hairball formation in cats with fibre is that fibre delays gastric emptying, leading to binding of single strands of hair to food particles, so that more hair is transferred into the duodenum and subsequently excreted untangled with the faeces.

It is notable that cellulose, on the other hand, did not induce any alteration to hairball formation. Both cellulose and sugarcane fibre are non-fermentable fibres that contain more than 90 % of insoluble fibre. When incubated with dog faecal inoculum, both fibre sources demonstrated negligible fermentation and gas production^(^^14^^)^. Thus, it is unlikely that solubility or fermentability differences among the fibre sources explain the results. Fibre length, however, was longer in sugarcane fibre than in cellulose (188 ± 1·8 *v.* 112 ± 1·7 µm), and this may explain the difference in their effects, as long fibres are believed to exert a stronger stimulus on peristalsis^(^^2^^)^.

We observed formation of large trichobezoars was greater in cats fed the CO diet containing low fibre and was reduced to zero in cats fed 20 % sugarcane fibre after 42 d of diet intake. Large trichobezoars are potentially the most harmful to cats because they are the most likely to induce abdominal discomfort, gastrointestinal alterations, anorexia, obstructions or vomiting in cats^(^^15^^,^^16^^)^. Thus, our study highlights the importance of the inclusion of dietary fibre in feline diets. In fact, the ingestion of plant material is part of the normal eating behaviour of cats, which has been previously reported in studies of cat intake of different plant material^(^^17^^,^^18^^)^.

The effect of sugarcane fibre on trichobezoar reduction was linear, suggesting that high inclusion amounts are necessary to achieve consistent effects on hairball prevention. However, it is important to consider that the present study evaluated only three fibre concentrations and other levels could be evaluated to find the optimum intake. In addition, not all diets can have high-fibre content, such as when a high-energy intake is desired. These are limitations that should be considered in future studies about hairballs in cats.

## Conclusion

Sugarcane fibre supplementation in feline dry kibble diets may reduce hairball formation. This suggests fibre supplementation may have a clinical application in cats for the prevention of health problems related to trichobezoars.
